# Dietary Risk Factors, Food Group Diversity, and Food Pattern Influences Assessed Using the Food Frequency Questionnaire on Cardiovascular Disease Risk and Other Noncommunicable Disease Profiles in Urban India

**DOI:** 10.7759/cureus.102950

**Published:** 2026-02-04

**Authors:** Jyoti Pradhan

**Affiliations:** 1 Department of Community Medicine and Family Medicine, All India Institute of Medical Sciences, Raipur, IND

**Keywords:** cardiovascular diseases, cardiovascular risk, dietary patterns, food frequency questionnaire, noncommunicable diseases (ncds), nutrition, who ish risk chart

## Abstract

Introduction: Evidence from the Global Burden of Disease 2023 study and the National Family Health Survey 5 of India suggests that diet is an important predictor of metabolic and cardiovascular diseases (CVDs). India, especially in the urban areas, is experiencing a rapid dietary transition marked by high consumption of refined carbohydrates and processed foods, which is contributing to the increasing burden of CVDs and related risk factors.

Objectives: This study aimed to quantify food group consumption patterns, average nutrient intake, and dietary diversity by the Food Frequency Questionnaire and explore their association with 10-year CVD risk by the World Health Organization International Society of Hypertension risk chart and other noncommunicable diseases like type II diabetes (by Indian Council of Medical Research and Research Society for the Study of Diabetes in India criteria) and hypertension (by Joint National Committee 8) in an urban adult population in Central India.

Methodology: Data were analyzed using IBM Statistical Package for the Social Sciences version 27 (IBM Corp., 2020, Armonk, NY). The association between food group frequency and CVD risk, diabetes, and hypertension was tested using the chi-square test. Significant variables were included in ordinal logistic regression for CVD scores and binary logistic regression for diabetes and hypertension. Correlations of CVD risk with average intake and dietary diversity were evaluated using Pearson’s test and linear regression as appropriate.

Results: A total of 400 participants were included in the study. The number of participants who consumed cereals daily was 400 (100%), other vegetables daily was 294 (73.5%), and green leafy vegetables weekly was 278 (69.5%), while intake of salad, fruits, nuts, milk, milk products, and nonvegetarian items was generally low or infrequent. Less frequent intake of salad (adjusted odds ratio (AOR) = 2.057, p = 0.001) and nuts/raisins (AOR = 1.868, p = 0.006) increased CVD risk, even after adjusting for behavioral factors like tobacco and alcohol. Twice-daily or more frequent tea was associated with diabetes (crude odds ratio (Crude OR) = 1.628, p = 0.023), which remained significant after adjusting for sociodemographic and behavioral variables. Less than weekly salad (Crude OR = 1.582, p = 0.029) and less than monthly milk product intake (Crude OR = 1.766, p = 0.005) were associated with hypertension. Increased quantity of salad was negatively correlated, and tea was positively correlated with CVD risk scores, with a p value of <0.05. Participants with higher dietary diversity had lower CVD risk scores (Adjusted R^2^ = 0.008, p = 0.037).

Conclusions: Daily consumption of salad/nuts/raisins is good for cardiovascular health. Rather than single foods, overall eating patterns and balanced diets play a greater role in chronic conditions.

## Introduction

Diet is a major, modifiable determinant of cardiometabolic risk. According to the dietary guidelines for Indians 2024, a balanced diet is paramount to prevent adverse effects of nutritional deficiency and ensure optimal growth and development of individuals. They further decrease the risk of noncommunicable diseases (NCDs) occurring in later life [1]. India has undergone a rapid dietary transition over the last two decades, with persistently high consumption of refined carbohydrates and an increasing intake of energy-dense processed, low-protein-quality foods, along with regional heterogeneity in macronutrient patterns. These trends are strongly associated with type II diabetes (T2DM), hypertension, obesity, and other cardiovascular disease (CVD) risk factors [2].

Global scenario

Globally, CVDs are the leading cause of death with an estimated 19.8 million deaths in 2022 in low- and middle-income countries [3]. While extensive studies have been conducted on the role of age, genetics, physical activity, and metabolic risk factors like dyslipidemia and insulin resistance on the onset of CVDs, the direct link between dietary consumption patterns and CVD events is an area of ongoing research [4]. Recent pooled analyses from the Global Burden of Disease study have highlighted that diets high in refined carbohydrates, sodium, and processed foods and low in fruits, vegetables, and whole grains substantially increase cardiovascular risk [5]. Dietary patterns rather than single nutrients are increasingly recognized as more relevant predictors of CVD outcomes. Mediterranean diets, Dietary Approaches to Stop Hypertension (DASH) diets, the Atkins diet, and plant-predominant diets are among the diets that have been evaluated to reduce mortality from NCDs and CVD risk worldwide [6-8]. However, no singular diet has yet been proven to be most effective for mitigating CVD risk.

Indian scenario

According to recent estimates, CVD accounts for more than 28% of all deaths in India, and the number of individuals at intermediate or high predicted 10-year CVD risk is increasing steadily [9]. India’s CVD epidemic is characterized by an earlier age of onset, rising prevalence, and large population-level burden that is driven by both conventional risk factors and population-level transitions in nutrition, demography, and environment [10]. The National Family Health Survey (NFHS) 5 India data show that 24% of women and 23% of men in India are overweight and obese [11]. It affects urban areas more than rural areas. At the same time, there is a chronic micronutrient deficiency in the population. Essential minerals such as iron, calcium, zinc, and magnesium are insufficiently present in our food. This phenomenon is called hidden hunger [12]. These micronutrients are essential for various integral functions in the human body. The Indian Council of Medical Research-Indian Diabetes (ICMR-INDIAB) survey (2025) reported persistently high intake of refined carbohydrates, low-quality fats, and inadequate protein, with marked regional variation across the country [2]. Urbanization has accelerated consumption of processed foods, sugary beverages, and restaurant-based diets, further aggravating obesity, hypertension, and T2DM. Media influence, with frequent advertising, has led to further expansion of fast-food culture. Urban Indians now allocate 11% of their monthly food expenditure to processed foods and beverages, surpassing spending on fruits and vegetables (Survey on household consumption expenditure India 2022-23) [13].

The Food Frequency Questionnaire (FFQ) is a widely used dietary assessment tool that captures habitual long-term food intake in epidemiological studies with reasonable validity and reproducibility [14]. The World Health Organization International Society of Hypertension (WHO ISH) nonlaboratory-based charts are globally validated charts, which are useful risk-prediction tools for future CVD events, especially in low-resource settings such as India. They are useful especially where laboratory testing is limited [15,16]. Although several FFQs have been adapted and validated for Indian populations, few community-based studies in India have combined FFQ-derived dietary assessment with WHO/ISH CVD risk prediction charts to assess how habitual diets translate into predicted CVD risk.

Thus, the objectives of this study were 1) to quantify the amount and variety of food groups consumed by the participants, 2) to estimate the average daily macronutrient and micronutrient intake from these foods, and 3) to find association between the 10-year future risk of CVD events and presence of other NCDs with current consumption patterns among adults in an urban area of India.

## Materials and methods

Study design and setting, and study population

This was a community-based, cross-sectional, analytical study conducted over two years (June 2022 to June 2024) in an urban area of Raipur, Chhattisgarh, India. The study population included adults aged >40 years residing in the urban field practice area of a tertiary care hospital in India for more than six months. Sample size was estimated from the study Singh et al. [17], using the formula $Z^2 p(1-p)/d^2$, taking the prevalence of moderate or high risk of future cardiovascular events as 54%, absolute precision 5%, and nonresponse rate of 10%, giving the minimum required sample size of 382. The final number of samples with complete dietary data collected was 400. The first street and first household were selected by lottery method, and subsequent houses were selected in a consecutive right direction. Only one adult was interviewed per household by the principal investigator. In households with more than one individual aged 40 years or older, only one individual was chosen by lottery method. The consent form and participant information sheet were administered to participants and explained in their local language.

Inclusion and exclusion criteria

Adults aged 40 years or older residing in the urban field practice area (Ward Nos. 24 and 25) of the All India Institute of Medical Sciences, Raipur, Chhattisgarh, India, who had been residing there for more than six months and were able to comprehend the questionnaire were included in the study. Individuals with a previous history of fatal CVDs or a history of previous cardiac surgeries were excluded from the study. Pregnant participants were also excluded from the study.

Study tools

A semistructured questionnaire, adapted from the WHO STEPwise approach to NCD risk factor surveillance survey, was utilized for data collection [18]. This incorporated sociodemographic details, addiction profile, history of NCDs, blood pressure, and other relevant measurements. The dietary component was adapted from the semiquantitative FFQ developed and validated for urban and rural Indian populations by Bowen et al. [14], complemented by nutrient values derived from the Indian Food Composition Tables 2017 and the Dietary Guidelines for Indians 2024 published by the ICMR-National Institute of Nutrition (NIN) [1]. The clarity and feasibility of the questionnaire were established through a pilot pretest among a small sample of participants to ensure comprehension, relevance, and practical usability. The feedback was incorporated prior to final administration. The complete questionnaires used for data collection are provided in Appendix 1.

Food frequency questionnaire

The FFQ used in this study was adapted from a semiquantitative FFQ developed and validated for urban and rural Indian populations by Bowen et al. [14]. It captures habitual dietary intake by recording the frequency and portion sizes of commonly consumed foods over a 12-month period. Individual eating patterns were estimated based on daily, weekly, and monthly consumption. For instance, a participant consuming a food item twice daily was recorded as "two times daily," while thrice-monthly consumption was recorded as "three times monthly." Foods consumed very infrequently or never were categorized under the "Less than Monthly" intake group. The food groups assessed included items recognized for both protective and harmful effects on cardiovascular health. A total of 12 food groups were included in the analysis.

Updated WHO ISH CVD risk chart

CVD risk was assessed using the 2020 WHO ISH nonlaboratory-based risk chart for Southeast Asian countries, which uses parameters such as age, gender, smoking status, systolic blood pressure, and body mass index (BMI) of participants. The CVD risk categories are as follows: green <5%, yellow 5% to <10%, orange 10% to <20%, red 20% to <30%, and deep red ≥30% [15].

Type II diabetes mellitus and hypertension

A digital sphygmomanometer was used to take the average blood pressure measurements from two readings. Participants were asked to have an empty bladder, refrain from speaking, and avoid coffee/tea before or during the measurements. The Joint National Committee (JNC 8) criteria were used to diagnose high blood pressure in individuals with no prior history of hypertension. New cases of T2DM were diagnosed based on capillary blood glucose measured by a glucometer in the community, following ICMR and Research Society for the Study of Diabetes in India (RSSDI) guidelines. The cutoffs for T2DM were as per ICMR and RSSDI guidelines, both of which use the standard diabetes cutoffs: fasting plasma glucose ≥126 mg/dL, HbA1c ≥6.5%, or random glucose ≥200 mg/dL with symptoms.

Statistical analysis

Data were entered into Microsoft Excel (Microsoft Corporation, Redmond, WA) and analyzed using IBM Statistical Package for the Social Sciences Statistics for Windows, version 27.0 (IBM Corp., 2020, Armonk, NY). The qualitative variables were expressed as frequencies and percentages. Continuous variables were reported as mean, median, standard deviation, and interquartile range. The association between the frequency of intake of different food groups and CVD risk categories, diabetes, and hypertension was tested using the chi-square test. A p value of <0.05 was taken as significant. The groups were tested to rule out multicollinearity. The food groups with significant association with CVD scores were further taken up for univariate and multivariate ordinal logistic regression, and those with diabetes and hypertension for binary logistic regression to estimate the crude odds ratio (Crude OR) and adjusted odds ratio (AOR). The results were adjusted in various models for sociodemographic variables like age, gender, religion, caste, socioeconomic status (SES), marital status, family type, and behavioral factors like smoking, smokeless tobacco, and alcohol intake. Goodness of fit, model fitting tests, and tests of parallel lines were also checked. The average daily macronutrient and macronutrient intake from various food groups for each individual were further compounded using nutrient charts from the Dietary Guidelines for Indians handbook by ICMR-NIN [1]. They were presented in box-and-whisker plots, showing the median and outliers. Pearson’s correlation test was used to assess the association between average nutrient intake and CVD risk scores. Those with a p value of <0.05 were further taken up for linear regression analysis to estimate the relative risk. Scatter plots were used to plot the correlation. Correlation of dietary diversity with CVD scores was measured using Pearson’s correlation.

## Results

Sociodemographic profile of the study participants

A total of 400 participants were included in the study. The sociodemographic profile of the study participants is given in Table 1. The average age of the participants was 52.51 ± 10.08 years. The median age was 50 years.

**Table 1:** Sociodemographic profile of the study participants ^*^Others include single/widowed/separated/divorced ^**^SES by the Updated Modified Kuppuswamy Scale [19] ^***^T2DM by history or capillary blood sugar using ICMR-RSSDI criteria; hypertension by history or by blood pressure measurement according to JNC 8 criteria OBC: other backward caste; SC/ST: scheduled caste/scheduled tribe; UR: unreserved; SES: socioeconomic status; T2DM: type 2 diabetes mellitus; ICMR: Indian Council of Medical Research; RSSDI: Research Society for the Study of Diabetes in India

Variables	Groups	Total (n = 400), n (%)
Age (years)	40-49	178 (44.5)
	50-59	98 (24.5)
	≥60	124 (31.0)
Gender	Female	225 (56.3)
	Male	175 (43.8)
Religion	Hindu	378 (94.5)
	Others	22 (5.5)
Caste	OBC	206 (51.5)
	SC/ST	79 (19.6)
	UR	115 (28.7)
Education	Illiterate	109 (27.3)
	Primary school	73 (18.3)
	Middle school	72 (18.0)
	High school	65 (16.3)
	Intermediate school/diploma	44 (11.0)
	Graduate and above	37 (9.3)
Occupation	Employed	241 (60.3)
	Unemployed	159 (39.8)
Marital status	Married	318 (79.5)
	Others^*^	82 (20.5)
SES^**^	Lower	8 (2.0)
	Upper lower	176 (44.0)
	Lower middle	136 (34.0)
	Upper middle	77 (19.3)
	Upper	3 (0.8)
Family type	Nuclear	212 (53.0)
	Extended	188 (47.0)
Smoking	Ever smoker	64 (16.0)
	Nonsmoker	336 (84.0)
Smokeless tobacco	Ever user	236 (59.0)
	Nonuser	164 (41.0)
Alcohol intake	Ever alcohol user	96 (24.0)
	Non-alcohol user	304 (76.0)
T2DM^***^	Diabetic	134 (33.5)
	Nondiabetic	266 (66.5)
Hypertension^***^	Yes	188 (47.0)
	No	212 (53.0)
Body mass index (kg/m^2^)	<20	61 (15.3)
	20-24	130 (32.5)
	25-29	154 (38.5)
	30-35	50 (12.5)
	≥35	5 (1.3)

Frequency of intake of food groups

Food Group Intake Frequency Pattern Among the Study Population Using FFQ

The frequency distribution of intake of all 12 food groups is given in Table 2. Information on the intake of nonvegetarian items, such as eggs, fish, or meat, was also collected.

**Table 2:** Frequency of food groups intake among the study participants using FFQ (n = 400) Salad was taken as the consumption of vegetables (tomato, cucumber, onion, beetroot, carrot, etc.) in raw form in a salad preparation. Green leafy vegetables and other vegetables are taken in cooked form. Fruits were consumed raw. Milk products include items like curd and paneer. Tea is standard tea with sugar. Cold/sweet drinks include soft drinks like Coca-Cola, store-bought juices, and sweetened drinks. Packaged items examples are biscuits like Parle G. Oily/fried items examples are potato-filled samosa. Cereals include rice/wheat/millets, etc. FFQ: Food Frequency Questionnaire

Food groups	Daily, n (%)	Weekly, n (%)	Monthly, n (%)	Less than monthly, n (%)
Salad	67 (16.8)	81 (20.3)	51 (12.8)	201 (50.2)
Green leafy vegetables	38 (9.5)	278 (69.5)	18 (4.5)	66 (16.5)
Other vegetables	294 (73.5)	93 (23.3)	3 (0.8)	10 (2.5)
Fruits	47 (11.8)	115 (28.7)	89 (22.3)	149 (37.3)
Nuts and raisins	28 (7.0)	40 (10.0)	44 (11.0)	288 (72.0)
Milk	22 (5.5)	29 (7.2)	46 (11.5)	303 (75.8)
Milk products	18 (4.5)	124 (31.0)	75 (18.8)	183 (45.8)
Tea	307 (76.8)	15 (3.8)	7 (1.8)	71 (17.8)
Cold/sweet drinks	14 (3.5)	51 (12.8)	61 (15.3)	274 (68.5)
Packaged items	92 (23.0)	125 (31.3)	44 (11.0)	139 (34.8)
Oily/fried items	24 (6.0)	123 (30.8)	97 (24.3)	156 (39.0)
Meat/fish/egg	3 (0.8)	208 (52.0)	64 (16.0)	125 (31.3)
Cereals	400 (100.0)	0 (0.0)	0 (0.0)	0 (0.0)

As shown in Table 2, salad intake was less than monthly in 201 (50.2%) participants. Green leafy vegetables were mostly eaten on a weekly basis by 278 (69.5%) participants. Other vegetables were consumed daily by the majority, that is, 294 (73.5%) of the participants. Fruit consumption was insufficient among the study population, with only 47 (11.8%) of people taking fruits daily. Similarly, the intake of nuts and raisins was also low. Very few participants consumed milk daily. The number of participants who never consumed milk was 303 (75.8%). Consumption of milk products, such as curd or paneer, was slightly higher than that of milk. About 124 (31.0%) of people consumed milk products weekly. The majority of the participants, about 307 (76.8%), consumed tea daily. Coffee was consumed by only a countable number of participants and hence was not taken into account. The frequency of tea intake varied from once to eight times a day, with a mode of two times per day. Cold drinks or sugar-sweetened drinks were consumed less frequently. About 274 (68.5%) participants did not take cold drinks regularly.

A high prevalence of intake of packaged food items was found. High consumption of oily or fried food items was also found, with 123 (30.8%) people taking it weekly and 97 (24.3%) taking it monthly. Finally, nonvegetarian food items like meat were consumed mostly on a weekly basis by 208 (52%) of participants. A total of 125 (31.3%) individuals were vegetarians. All the participants in the study consumed some kind of cereal as a staple food daily.

Association of Food Group Intake Frequency Pattern With CVD Risk Scores

For aid in further analysis of the study, the orange, red, and deep red categories were taken as one category. The CVD risk categorization is as follows: green (<5%) minimal risk, yellow (5% to <10%) mild risk, and orange and above categories (≥10%) moderate and high risk. After chi-square tests for association, lower intake of salad and nuts was significantly associated with an increased future risk of CVD, as shown in Table 3.

**Table 3:** Association of food intake pattern with CVD risk categories using WHO ISH risk chart ^*^Significant p < 0.05 by chi-square test CVD: cardiovascular disease; WHO ISH: World Health Organization International Society of Hypertension

Variable (food item intake)	Groups	CVD risk categories, n (%)			Total (n = 400), n (%)	Chi-square (df)	p value
		Green (n = 201) (<5%)	Yellow (n = 117) (5% to <10%)	Orange/red (n = 82) (≥10%)			
Salad	Less than weekly	113 (56.2)	73 (62.4)	66 (80.5)	252 (63.0)	14.742 (2)	0.001^*^
	Daily and weekly	88 (43.8)	44 (37.6)	16 (19.5)	148 (37.0)		
Green leafy vegetables	Less than daily	179 (89.1)	110 (94.0)	73 (89.0)	362 (90.5)	2.379 (2)	0.304
	Daily	22 (10.9)	7 (6.0)	9 (11.0)	38 (9.5)		
Other vegetables	Less than twice daily	135 (67.2)	72 (61.5)	47 (57.3)	254 (63.5)	2.711 (2)	0.258
	Twice daily or more	66 (32.8)	45 (38.5)	35 (42.7)	146 (36.5)		
Fruits	No fruit daily	173 (86.1)	108 (92.3)	72 (87.8)	353 (88.3)	2.795 (2)	0.247
	Fruit daily	28 (13.9)	9 (7.7)	10 (12.2)	47 (11.8)		
Nuts/raisins	Less than monthly	133 (66.2)	88 (75.2)	67 (81.7)	288 (72.0)	7.822 (2)	0.020^*^
	Daily/weekly/monthly	68 (33.8)	29 (24.8)	15 (18.3)	112 (28.0)		
Milk	Less than monthly	151 (75.1)	89 (76.1)	63 (76.8)	303 (75.8)	0.101 (2)	0.951
	Daily/weekly/monthly	50 (24.9)	28 (23.9)	19 (23.2)	97 (24.3)		
Milk products	Less than monthly	84 (41.8)	56 (47.9)	43 (52.4)	183 (45.8)	2.958 (2)	0.228
	Daily/weekly/monthly	117 (58.2)	61 (52.1)	39 (47.6)	217 (54.3)		
Meat/fish/eggs	Monthly or less	93 (46.3)	60 (51.3)	36 (43.9)	189 (47.3)	1.209 (2)	0.546
	Daily and weekly	108 (53.7)	57 (48.7)	46 (56.1)	211 (52.8)		
Tea	Daily twice or more	105 (52.2)	67 (57.3)	54 (65.9)	226 (56.5)	4.432 (2)	0.109
	Once daily or less	96 (47.8)	50 (42.7)	28 (34.1)	174 (43.5)		
Cold/sweet drinks	Daily/weekly	35 (17.4)	22 (18.8)	8 (9.8)	65 (16.3)	3.301 (2)	0.2
	Monthly and less than monthly	166 (82.6)	95 (81.2)	74 (90.2)	335 (83.8)		
Packaged items	Daily	42 (20.9)	24 (20.5)	26 (31.7)	92 (23.0)	4.422 (2)	0.11
	Less than daily	159 (79.1)	93 (79.5)	56 (68.3)	308 (77.0)		
Oily/fried food	Monthly twice or more	119 (59.2)	70 (59.8)	42 (51.2)	231 (57.8)	1.815 (2)	0.404
	Monthly once or less	82 (40.8)	47 (40.2)	40 (48.8)	169 (42.3)		

All those variables that had p values of <0.2 in the previous table were considered for ordinal logistic regression in Table 4.

**Table 4:** Comparison of crude OR and AOR of CVD risk categories with food intake frequency pattern ^*^Significant p < 0.05 by ordinal logistic regression Model 1 is unadjusted (univariate ordinal logistic regression), Model 2 is adjusted for age, gender, religion, caste, SES, marital status, and family type, with all four food groups separately. Model 3 is adjusted for behavioral factors such as smokeless tobacco and alcohol for all four food groups separately with CVD risk categories (multivariate ordinal logistic regression) WHO ISH: World Health Organization International Society of Hypertension; CVD: cardiovascular disease; Crude OR: crude odds ratio; CI: confidence interval; AOR: adjusted odds ratio; SES: socioeconomic status

Variable (food item intake)	Groups	WHO ISH CVD risk categories					
		Model 1		Model 2		Model 3	
		Crude OR (95% CI)	p value	AOR (95% CI)	p value	AOR (95% CI)	p value
Salad	Less than weekly	2.010 (0.302-1.095)	0.001^*^	1.534 (-0.106 to 0.963)	0.116	2.022 (0.297-1.111)	0.001^*^
	Daily and weekly	1		1		-	
Nuts/raisins	Less than monthly	1.835 (0.178-1.036)	0.006^*^	1.284 (-0.337 to 0.837)	0.404	1.772 (0.136 to 1.007)	0.010^*^
	Daily/weekly/monthly	1		1		1	
Tea	Once daily or less	0.678 (-0.766 to -0.011)	0.044^*^	0.680 (-0.882 to 0.113)	0.130	0.798 (-0.613 to 0.161)	0.252
	Daily twice or more	1		1		1	
Packaged items	Less than daily	0.701 (-0.790 to 0.081)	0.110	0.688 (-0.962 to 0.215)	0.213	0.687 (-0.817 to -0.065)	0.095
	Daily	1		1		1	

As shown in Table 4, participants with less than weekly salad intake and less than monthly nuts/raisins intake had higher odds of being in higher WHO ISH CVD risk categories in the univariate model, which remained significant when adjusted for behavioral factors like intake of tobacco and alcohol in the multivariate model. However, the association was not significant when adjusted for sociodemographic factors.

The association of tea intake with CVD risk categories was significant only in the univariate model, while less than daily intake of packaged items like biscuits was not significantly associated.

Association of Food Group Intake Frequency Pattern With T2DM and Hypertension

Using the chi-square test of significance, the daily intake pattern of tea was found to be significantly associated with the presence of T2DM. The rest of the variables were insignificant, as shown in Table 5.

**Table 5:** Association of food intake pattern with T2DM ^*^Significant p < 0.05 by chi-square test T2DM: type 2 diabetes mellitus

Variable (food item intake)	Groups	Type II diabetes mellitus, n (%)		Total (n = 400), n (%)	Chi-square	p value
		Yes (n = 134)	No (n = 266)			
Salad	Less than weekly	92 (68.7)	160 (60.2)	252 (63.0)	2.766	0.096
	Daily and weekly	42 (31.3)	106 (39.8)	148 (37.0)		
Green leafy vegetables	Less than daily	122 (91.0)	240 (90.2)	362 (90.5)	0.07	0.792
	Daily	12 (9.0)	26 (9.8)	38 (9.5)		
Other vegetables	Less than twice daily	86 (64.2)	168 (63.2)	254 (63.5)	0.04	0.841
	Twice daily or more	48 (35.8)	98 (36.8)	146 (36.5)		
Fruits	No fruit daily	122 (91.0)	231 (86.8)	353 (88.3)	1.518	0.218
	Fruit daily	12 (9.0)	35 (13.2)	47 (11.8)		
Nuts/raisins	Less than monthly	95 (70.9)	193 (72.6)	288 (72.0)	0.122	0.727
	Daily/weekly/monthly	39 (29.1)	73 (27.4)	112 (28.0)		
Milk	Less than monthly	96 (71.6)	207 (77.8)	303 (75.8)	1.851	0.174
	Daily/weekly/monthly	38 (28.4)	59 (22.2)	97 (24.3)		
Milk products	Less than monthly	55 (41.0)	128 (48.1)	183 (45.8)	1.797	0.180
	Daily/weekly/monthly	79 (59.0)	138 (51.9)	217 (54.3)		
Meat/fish/eggs	Monthly or less	64 (47.8)	125 (47.0)	189 (47.3)	0.021	0.884
	Daily and weekly	70 (52.2)	141 (53.0)	211 (52.8)		
Tea	Daily twice or more	65 (48.5)	161 (60.5)	226 (56.5)	5.237	0.022^*^
	Once daily or less	69 (51.5)	105 (39.5)	174 (43.5)		
Cold/sweet drinks	Daily/weekly	21 (15.7)	44 (16.5)	65 (16.3)	0.05	0.824
	Monthly and less than monthly	113 (84.3)	222 (83.5)	335 (83.8)		
Packaged items	Daily	30 (22.4)	62 (23.3)	92 (23.0)	0.043	0.836
	Less than daily	104 (77.6)	204 (76.7)	308 (77.0)		
Oily/fried food	Monthly twice or more	80 (59.7)	151 (56.8)	231 (57.8)	0.315	0.575
	Monthly once or less	54 (40.3)	115 (43.2)	169 (42.3)		

The Crude OR of tea intake with T2DM using binary logistic regression was 1.628 (95% CI = 1.071-2.474, p = 0.023). Thus, participants taking tea twice daily or more had 1.628 times more risk of having T2DM than those who took it in lesser frequencies.

This association remained significant when adjusted for age, gender, religion, caste, SES, marital status, family type, smoking, smokeless tobacco, and alcohol, and also with the rest of the food variables (Table 6).

**Table 6:** Influence of tea consumption patterns with the odds of T2DM ^*^Significant p < 0.05 using binary logistic regression Model 1 is unadjusted, Model 2 is adjusted for age, gender, religion, caste, SES, marital status, and family type, Model 3 is adjusted for smoking and smokeless tobacco, and alcohol in addition to Model 2 factors. Model 4 is adjusted for Model 3 factors plus the rest of the food item intake variables Crude OR: crude odds ratio; CI: confidence interval; AOR: adjusted odds ratio; SES: socioeconomic status; T2DM: type 2 diabetes mellitus

Variable (food item intake)	Groups	Type II diabetes mellitus							
		Model 1		Model 2		Model 3		Model 4	
		Crude OR (95% CI)	p value	AOR (95% CI)	p value	AOR (95% CI)	p value	AOR (95% CI)	p value
Tea	Daily twice or more	1.628 (1.071-2.474)	0.023^*^	1.618 (1.041-2.514)	0.032^*^	1.692 (1.081-2.650)	0.022^*^	1.715 (1.078-2.728)	0.023^*^
	Once daily or less	1		1		1		1	

Using chi-square, intake patterns of salad and milk products were found to be significantly associated with the presence of hypertension, with p values <0.05 (Table 7).

**Table 7:** Association of food intake pattern with hypertension ^*^Significant p < 0.05 by chi-square test

Variable (food item intake)	Groups	Hypertension, n (%)		Total (n = 400), n (%)	Chi-square	p value
		Yes (n = 188)	No (n = 212)			
Salad	Less than weekly	129 (68.6)	123 (58.0)	252 (63.0)	4.801	0.028^*^
	Daily and weekly	59 (31.4)	89 (42.0)	148 (37.0)		
Green leafy vegetables	Less than daily	172 (91.5)	190 (89.6)	362 (90.5)	0.404	0.525
	Daily	16 (8.5)	22 (10.4)	38 (9.5)		
Other vegetables	Less than twice daily	119 (63.3)	135 (63.7)	254 (63.5)	0.006	0.937
	Twice daily or more	69 (36.7)	77 (36.3)	146 (36.5)		
Fruits	No fruit daily	170 (90.4)	183 (86.3)	353 (88.3)	1.619	0.203
	Fruit daily	18 (9.6)	29 (13.7)	47 (11.8)		
Nuts/raisins	Less than monthly	143 (76.1)	145 (68.4)	288 (72.0)	2.906	0.088
	Daily/weekly/monthly	45 (23.9)	67 (31.6)	112 (28.0)		
Milk	Less than monthly	143 (76.1)	160 (75.5)	303 (75.8)	0.019	0.89
	Daily/weekly/monthly	45 (23.9)	52 (24.5)	97 (24.3)		
Milk products	Less than monthly	100 (53.2)	83 (39.2)	183 (45.8)	7.914	0.005^*^
	Daily/weekly/monthly	88 (46.8)	129 (60.8)	217 (54.3)		
Meat/fish/eggs	Monthly or less	93 (49.5)	96 (45.3)	189 (47.3)	0.7	0.403
	Daily and weekly	95 (50.5)	116 (54.7)	211 (52.8)		
Tea	Daily twice or more	112 (59.6)	114 (53.8)	226 (56.5)	1.364	0.243
	Once daily or less	76 (40.4)	98 (46.2)	174 (43.5)		
Cold/sweet drinks	Daily/weekly	33 (17.6)	32 (15.1)	65 (16.3)	0.443	0.506
	Monthly and less than monthly	155 (82.4)	180 (84.9)	335 (83.8)		
Packaged items	Daily	49 (26.1)	43 (20.3)	92 (23.0)	1.88	0.17
	Less than daily	139 (73.9)	169 (79.7)	308 (77.0)		
Oily/fried food	Monthly twice or more	109 (58.0)	122 (57.5)	231 (57.8)	0.008	0.931
	Monthly once or less	79 (42.0)	90 (42.5)	169 (42.3)		

The Crude ORs of association of hypertension with salad (less than weekly intake) were 1.582 (95% CI = 1.048-2.387, p = 0.029) and with milk products (less than monthly intake) were 1.766 (95% CI = 1.187-2.629, p = 0.005). The association of milk product consumption pattern with hypertension remained significant when adjusted for age, gender, religion, caste, SES, marital status, family type, smoking, smokeless tobacco, and alcohol, and also with the rest of the food variables. But for the salad, it was not significant, as given in Table 8.

**Table 8:** Influence of salad and milk products consumption patterns on the odds of hypertension ^*^Significant p < 0.05 using binary logistic regression Model 1 is unadjusted, Model 2 is adjusted for age, gender, religion, caste, SES, marital status, and family type for salad and milk products separately, Model 3 is adjusted for smoking, smokeless tobacco, and alcohol in addition to Model 3 factors for salad and milk products separately with hypertension. Model 4 is adjusted for Model 3 factors plus all 12 food intake variables Crude OR: crude odds ratio; CI: confidence interval; AOR: adjusted odds ratio; SES: socioeconomic status

Variable (food item intake)	Groups	Hypertension							
		Model 1		Model 2		Model 3		Model 4	
		Crude OR (95% CI)	p value	AOR (95% CI)	p value	AOR (95% CI)	p value	AOR (95% CI)	p value
Salad	Less than weekly	1.582 (1.048-2.387)	0.029^*^	1.408 (0.900-2.201)	0.134	1.413 (0.902-2.215)	0.131	1.204 (0.747-1.942)	0.446
	Daily and weekly	1		-		1		1	
Milk products	Less than monthly	1.766 (1.187-2.629)	0.005^*^	1.823 (1.191-2.790)	0.006*	1.874 (1.219-2.880)	0.004^*^	1.952 (1.224-3.112)	0.005*
	Daily/weekly/monthly	1		1		1		1	

Amount of intake of food groups

Amount of Daily Intake of Different Food Groups Among the Study Population

**Figure 1:**
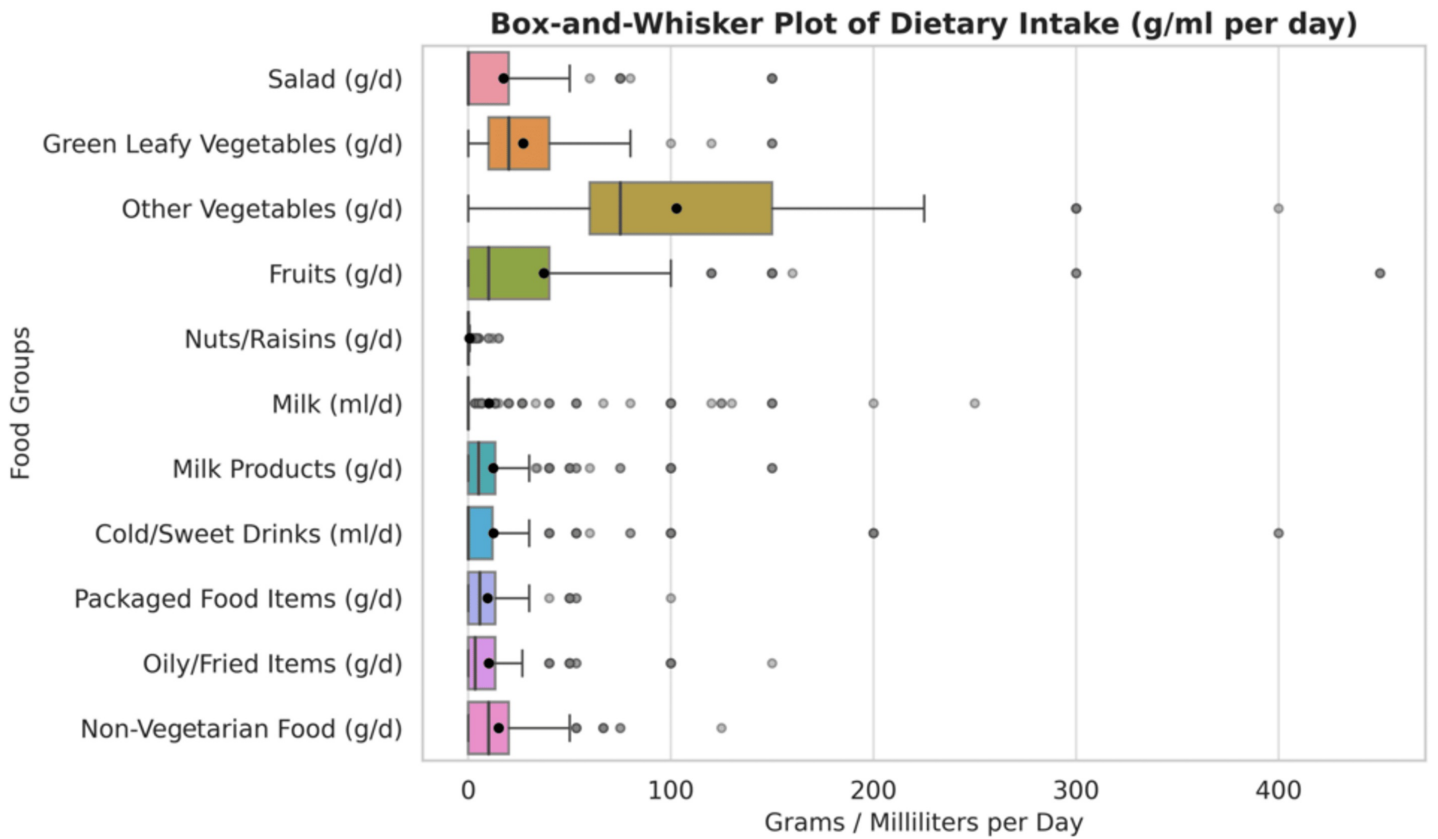
Horizontal box-and-whisker plot of daily dietary intake of food items from different food groups Each row represents the distribution of intake (in grams or milliliters per day) for a different food category across the dataset

Figure 1 shows the horizontal box-and-whisker plot for daily dietary intake from different food groups. In our study (as shown in the box-and-whisker plot), daily intake was highest for vegetables and fruits, with wide variability in their consumption. Milk, milk products, and nonvegetarian foods were consumed in small to moderate amounts with many low-intake outliers. Packaged, oily, and sweet drink items had low median intakes but numerous high-value outliers, indicating occasional high consumption among some participants.

Average Nutritional Value Obtained From Each Food Group

The average daily nutritional values of macronutrients and micronutrients obtained from each food group are given in Appendix 2.

Correlation of Daily Nutritional Value Obtained From Different Food Groups With CVD Risk Scores

Taking average daily consumption of food items as continuous variables, Pearson’s correlation was used to test for their correlation with WHO ISH CVD risk scores, as shown in Table 9.

**Table 9:** Correlation between average daily intake of different food groups with WHO ISH CVD risk scores ^*^Significant p < 0.05 by Pearson correlation coefficient WHO ISH: World Health Organization International Society of Hypertension; CVD: cardiovascular disease

Variable	WHO ISH CVD risk scores	
	Correlation coefficient	p value
Salad (g/d)	-0.121	0.015^*^
Green leafy vegetables (g/d)	-0.024	0.632
Other vegetables (g/d)	0.095	0.057
Fruits (g/d)	0.013	0.801
Nuts/raisins (g/d)	-0.067	0.182
Milk (mL/d)	0.033	0.516
Milk products (g/d)	0.035	0.480
Tea (mL/d)	0.136	0.006^*^
Cold/sweet drinks (mL/d)	0.027	0.592
Packaged food items (g/d)	-0.013	0.788
Oily/fried items (g/d)	0.058	0.247
Nonvegetarian items (g/d)	0.016	0.756

**Figure 2:**
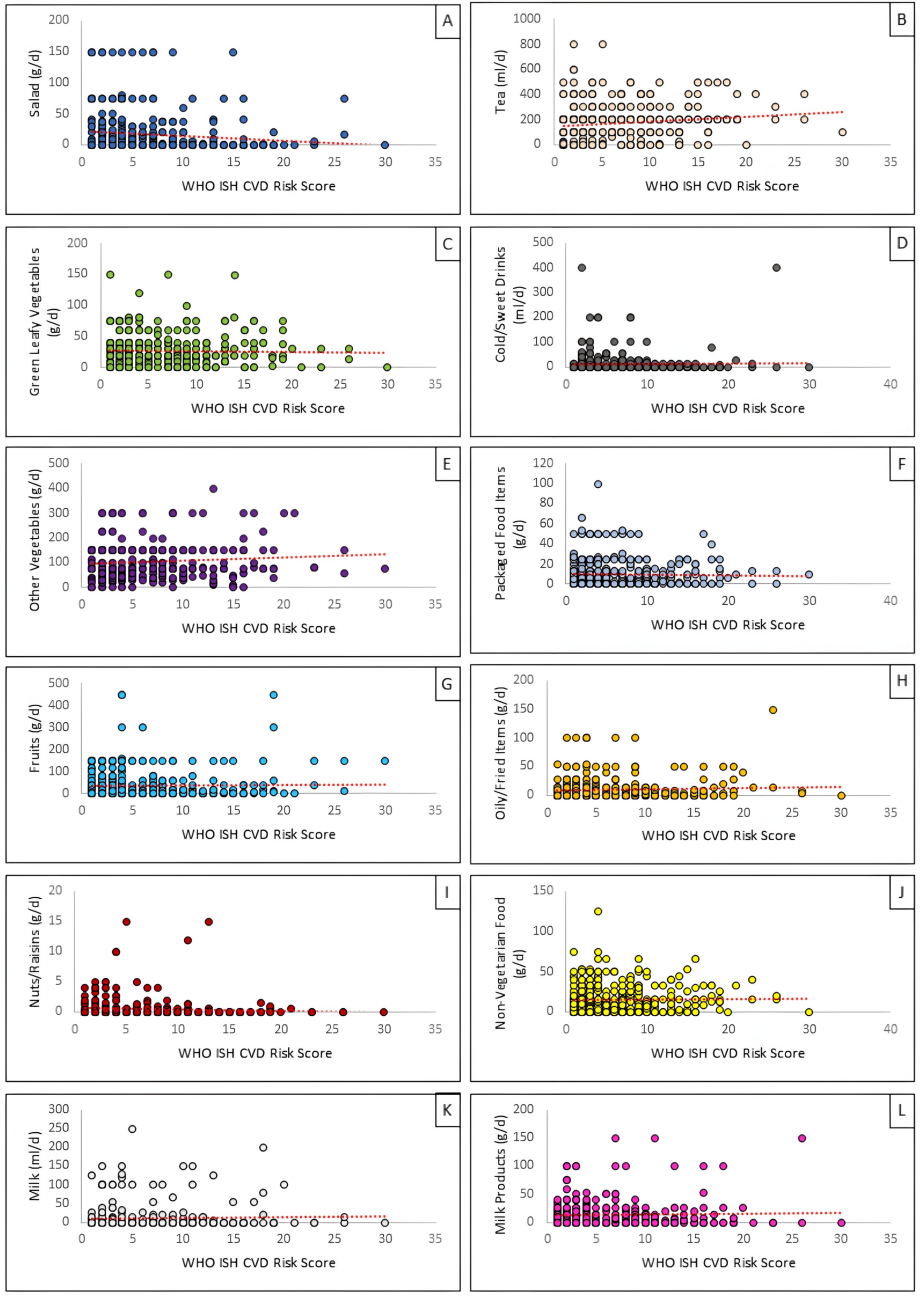
Linear relationship between WHO ISH CVD risk scores with average daily intake of different food groups (A) Salad (g/d), (B) tea (mL/d), (C) green leafy vegetables (g/d), (D) cold/sweet drinks (mL/d), (E) other vegetables (g/d), (F) packaged food items (g/d), (G) fruits (g/d), (H) oily/fried items (g/d), (I) nuts/raisins (g/d), (J) nonvegetarian food like meat/fish/eggs (g/d), (K) milk (mL/d), and (L) milk products (g/d) WHO ISH: World Health Organization International Society of Hypertension; CVD: cardiovascular disease

Scatter plots, where the daily average consumption of the participants was used as the dependent variable on the Y-axis, and WHO ISH CVD risk scores were plotted on the X-axis, are shown in Figure 2.

Salad and tea are the variables in the correlation table with p values <0.05 and were considered further for linear regression analysis, as shown in Table 10.

**Table 10:** Comparison of crude and adjusted risk ratios of WHO ISH CVD risk scores with average daily intake of different food groups ^*^Significant by linear regression analysis, p < 0.05, Model 1 is unadjusted (crude) ^**^Model 2 adjusted for both salad intake and tea intake of the participants ^***^Model 3 adjusted for salad intake, tea intake, other vegetables, and nuts/raisins WHO ISH: World Health Organization International Society of Hypertension; CVD: cardiovascular disease; CI: confidence interval

Variables	WHO ISH CVD risk scores								
	Model 1 (unadjusted)^*^			Model 2 (adjusted)^**^			Model 3 (adjusted)^***^		
	Adjusted R^2^	95% CI	p value	Adjusted R^2^	95% CI	p value	Adjusted R^2^	95% CI	p value
Salad^**^	0.015	-0.034 to -0.004	0.015	0.036	-0.036 to -0.006	0.007^*^	0.045	-0.035 to -0.004	0.015^*^
Tea^***^	0.018	0.001 to 0.008	0.006^*^		0.002 to 0.009	0.003^*^		0.002 to 0.009	0.005^*^

In the unadjusted Model 1, salad with F (1, 398) = 5.954, p = 0.015, and tea intake with F (1, 398) = 7.502, p = 0.006, are given. Tea was found to be a significant predictor of WHO ISH risk scores. In Model 2, both salad and tea were considered together and were found to be significant predictors of WHO ISH risk scores. Thus, 3.6% of the changes in WHO ISH risk scores could be explained by salad and tea considered together in model 2, with F (2,397) =7.425, p = 0.001.

In Model 3, the p values still remain significant after being adjusted with other food variables, with p values <0.2. The model is significant (from the ANOVA table) with F (4, 395) = 4.661, p = 0.001. Adjusted R^2^ is 4.5%. Therefore, 4.5% of the variance in the dependent variable, that is, WHO ISH Risk Scores, is explained by the predictor variables in Model 3.

Food diversity

**Figure 3:**
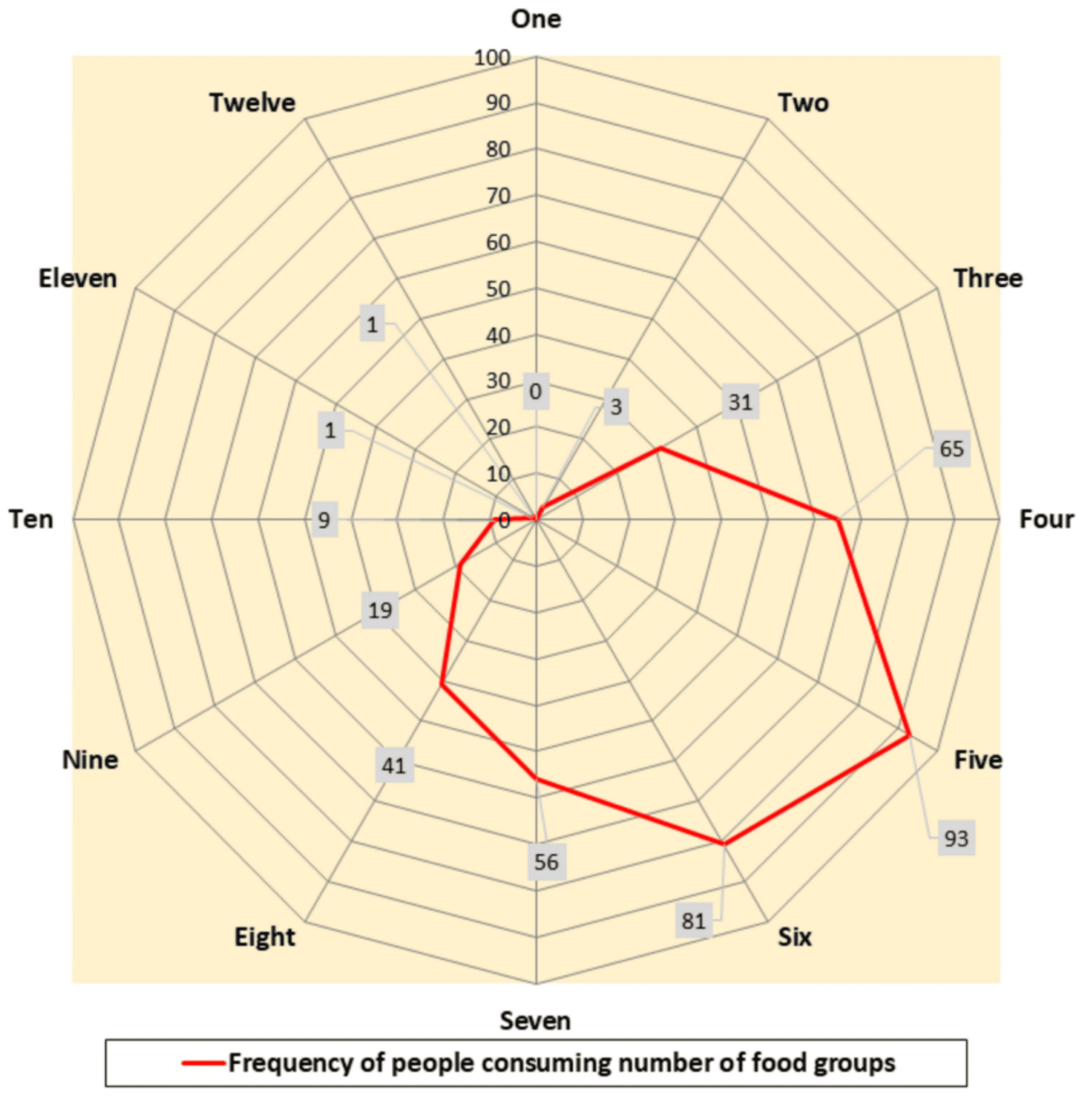
Food intake diversity among the study participants (n = 400) Twelve food groups included to estimate dietary diversity are salad/roots, green leafy vegetables, other vegetables, fruits, nuts and raisins, milk, other milk products, meat/fish/eggs, sweet drinks, oily food, cereals, and miscellaneous food items, such as packed foods and biscuits

Dietary diversity refers to the variety of food groups consumed over a given period and serves as a proxy for overall diet quality. Higher dietary diversity is linked to better nutrient adequacy and reduced risk of NCDs, including CVD. Dietary diversity is usually calculated based on the number of food groups consumed by an individual over a given recall period (commonly 24 hours or seven days). The Dietary Diversity Score is the count of food groups consumed, regardless of portion size or frequency (though some indices also set a minimum amount, e.g., ≥15 g). The majority of the participants in the current study consumed less than or equal to six food groups in a day, as shown in Figure 3.

Correlation of Dietary Diversity With CVD Scores

The dietary diversity of the participants, that is, the number of food groups consumed per day, was negatively correlated with WHO ISH CVD scores with r = -0.104, p = 0.037 (significant p <0.05), using the Pearson correlation coefficient. Adjusted R^2^ was 0.008 following linear regression with a p value of 0.037. Thus, as the number of food groups consumed per day increased, the WHO ISH CVD scores decreased.

## Discussion

The current study employs FFQ to quantify food group consumption frequency and amounts. A 2024 study in North Karnataka, India, found a strong correlation between nutrient intakes estimated from FFQ and those from 24-hour recalls and recommended that FFQ was valid and reliable for large-scale epidemiological studies [20]. The findings from our study showed that the daily intake frequency was highest for cereals, vegetables, and tea. Milk, milk products, and nonvegetarian foods were consumed in small to moderate amounts with many low-intake outliers. Packaged, oily, and sweet drink items had low median intakes but numerous high-value outliers, indicating occasional high consumption among some participants.

Our study included a large number of participants from urban slum areas, with 71.1% participants belonging to OBC or SC/ST categories and with high unemployment and limited literacy, which could have been a factor in the inability of the majority of the participants to afford high-quality food like fruits or nuts and raisins, which can be expensive. During the field experience, it was observed that processed and packaged food items, such as samosas, kachoris, and biscuits, are often more readily available to individuals, particularly in urban communities of lower economic strata. Hence, urban communities suffer from malnutrition more frequently than their rural counterparts, where an agrarian culture ensures the availability of fruits, milk, and vegetables at lower prices. This finding is similar to the one found in the ICMR-INDIAB survey [2].

Frequency intake pattern and association with CVD risk scores

Our study found that less frequent intake of salad and nuts was strongly associated with higher CVD risk categories, as assessed by the WHO ISH risk chart, even after adjusting for other dietary and lifestyle factors. The multivariate analysis suggests that salad/nuts/raisins consumption may be an independent predictor of cardiovascular health.

Dietary assessments using FFQs across regions in India and internationally have consistently reported low intakes of fruits, vegetables, and nuts among high-risk groups for coronary heart disease [8,21,22]. Wang et al., in the Chinese Longitudinal Healthy Longevity Survey, reported a 16% lower risk of CVD among adults who consumed the most fruits and vegetables compared with those who consumed the least [23]. High consumption of nuts and seeds (about 30 g/day) is associated with a ~20% lower risk of CVD and coronary heart disease, mainly through modest reductions in total and low-density lipoprotein cholesterol, while showing no significant effect on type 2 diabetes or blood pressure [22].

The lack of significant association for other food groups, such as milk and milk products, may be due to the low daily intake observed in the study population, which limits the statistical power to detect associations.

Frequency intake pattern and association with T2DM and hypertension

A daily twice or more intake pattern of tea was found to be significantly associated with the presence of T2DM. While moderate consumption of specific tea, such as unsweetened green tea or black tea, may have potential benefits in improving insulin sensitivity in diabetes [24], the population in our study mostly had intake of tea with milk and sugar, which could have been a contributing factor in T2DM. Further, in a 15-year prospective cohort of 5,248 Iranian adults, those in the highest tea-intake group had a significantly higher risk of acute myocardial infarction compared to those with the lowest tea intake [25].

Participants who took salad less than weekly and milk products less than monthly had a higher prevalence of hypertension. This is similar to the DASH diet recommendations for hypertension [7]. A study in Japan also reported that higher yogurt consumption was associated with lower BMI, reduced HbA1c, lower triglycerides, and higher high-density lipoprotein-cholesterol, indicating a healthier CVD profile [26].

Sakir et al. showed that foods high in fat, sugar, and sodium were strongly associated with the risks of obesity, T2DM, and hypertension, underscoring the importance of moderating the intake frequency of such foods for metabolic health [27]. Gay et al. analyzed the original DASH trial and found that both the DASH and fruits and vegetables diets reduced the 10-year atherosclerotic CVD risk by about 10%, with even greater benefits for women and Black participants. This is consistent with extensive evidence that the DASH diet, which emphasizes fruits, vegetables, whole grains, nuts, and low-fat dairy while limiting saturated fat and processed foods, effectively reduces cardiovascular risk and improves cardiometabolic outcomes [28].

Average nutritional intake amount and association with CVD risk scores

In our study, the highest daily intake was observed for vegetables and fruits, but the variability in consumption was wide, indicating inconsistent dietary habits. The box-and-whisker plots revealed that milk, milk products, and nonvegetarian foods were consumed in smaller amounts, with many outliers, suggesting that some individuals may be at risk of nutrient deficiencies. Similar to the dietary frequency pattern, a higher average daily intake of salad was negatively correlated with CVD scores, whereas an increased amount of tea was correlated with higher CVD scores.

An extensive UK Biobank study by Wang et al. found that higher adherence to a healthy, predominantly plant-based diet, incorporating nonstarchy vegetables, fruits, whole grains, fish, eggs, and low-fat dairy, was associated with significantly reduced incidence and mortality of CVD over a median follow-up of 12.3 years [8].

Dietary diversity vs. CVD risk scores

Regarding dietary diversity, a more varied diet is associated with a lower risk of CVD. This is supported by previous research showing that dietary diversity is a proxy for overall diet quality and is linked to better nutrient adequacy and reduced risk of NCDs. The negative correlation between dietary diversity and CVD risk scores remained significant after adjustment for other factors, indicating that dietary diversity may be an independent predictor of cardiovascular health. Higher ultraprocessed food intake was linked to lower dietary diversity and greater micronutrient inadequacy in another Indian study [29]. Similar results were obtained from a study in Thailand on community-dwelling older adults [30]. The NFHS-5 survey also found that greater dietary diversity was inversely associated with multiple NCDs, which are key CVD factors [11].

Strengths

This was a community-based study that also included participants from urban slum areas, which strengthens the representativeness of the sample and enhances the generalizability of the findings. Additionally, the use of an FFQ provided a more accurate and concise assessment of habitual dietary intake compared to a single 24-hour recall.

Limitations

Since the study is cross-sectional, temporal association could not be established. There are better risk prediction tools for CVD, such as Framingham risk scores, which use more expensive laboratory tests that are not feasible in the community. We considered only the adult population aged more than 40 years. The study may be subject to recall bias due to self-reported dietary intake and reporting bias, including social desirability bias related to lifestyle behaviors. Other variables, like physical activity and sleep, can be included in the study. FFQ is not as good as other meticulous methods of dietary assessment, such as the weighment method. FFQ tends to overestimate food and nutrient intake compared with 24-hour recalls, although its validity remains high [20].

## Conclusions

Simple dietary choices, such as including salads regularly, could have meaningful impacts on heart health. Salad is an important dietary intervention to reduce the risk of future CVDs and NCDs that can be easily instituted in the population. Policies and interventions aimed at increasing the variety of foods consumed, particularly vegetables, fruits, nuts, and whole grains, may be effective in improving CVD outcomes in the population. Promoting dietary diversity is important to reduce CVD risk. The community dietary intervention must be three-pronged, emphasizing not only the quantity of food but also its quality and diversity. Sourcing from naturally available products and imposing price controls on market produce of quality vegetables, fruits, and nuts is essential. Along with that, there is a need to create awareness among the population to eschew easily available, unhealthy, high-calorie foods and beverages. The study was followed by information, education, and communication programs to educate the people about the beneficial effects of various seasonal fruits and vegetables and also to avoid harmful food items. Rather than single foods, overall eating patterns and balanced diets play a larger role in managing chronic conditions.

## Appendices

Appendix 1

The questionnaires used in the study are as follows.

**Table 11:** Questionnaire Part I (sociodemographic, addiction, past disease history and measurements) The semistructured questionnaire used for data collection was adapted by our team from the World Health Organization STEPwise approach to Surveillance to the NCD risk factor survey [18]. The questionnaire was pilot tested to ensure clarity and feasibility INR: Indian rupee; WHO ISH: World Health Organization International Society of Hypertension; CVD: cardiovascular disease; NCD: noncommunicable disease; WHO STEPS: World Health Organization STEPwise approach to Surveillance

Question no.	Questions	Responses
	Participant ID (confidential)	
	Date of interview	
A.	Sociodemographic details	
A1.	Patient name (Confidential)	
A2.	Age (DOB) (≥40 and ≤74)	
A3.	Gender	Male; Female; Other
A4.	Address	
A5.	Religion	
A6.	Caste	
A7.	Education	
A8.	Occupation	
A9.	Marital status	Married; Single; Separated; Divorced; Widowed
A10.	Education of head of the family (with coding)	Code Illiterate (1); Primary school (2); Middle school (3); High school (4); Intermediate or post-high school diploma (5); Graduation (6); Professional course completed/Honors (7)
A11.	Occupation of head of the family (with coding)	Profession (10); Semi-profession (6); Clerical, shop-owner, farmer (5); Skilled worker (4); Semi-skilled worker (3); Unskilled worker (2); Unemployed (1)
A12.	Total income of the family per month (in INR)	
A13.	Socioeconomic class (as per Updated Modified Kuppuswamy Scale)	Lower (<5); Upper Lower (5-10); Lower Middle (11-15); Upper Middle (16-25); Upper (26-29)
A14.	Type of family	Nuclear; Extended; Others (specify)
B.	Tobacco and alcohol use	
B1.	Do you currently smoke any tobacco products, such as cigarettes, cigars, pipes, and waterpipe with tobacco?	Yes No
B2.	Did you smoke in the past (but not now)?	Yes No
B3.	Do you currently use any smokeless tobacco such as (chewing tobacco, betel, snuff)?	Yes No
B4.	Did you use smokeless tobacco in the past (but not now)?	Yes No
B5.	Do you currently consume an alcoholic drink?	Yes No
B6.	Did you consume alcohol products in the past (but not now)?	Yes No
C.	Past history	
C1.	Do you have type II diabetes mellitus?	Yes; If yes, since how many years? No
C2.	Do you have hypertension?	Yes; If yes, since how many years? No
C3.	Do you have any other chronic illness?	Yes; If yes, since how many years? No
D.	Measurements	
D1.	Weight (in kg)	
D2.	Height (in kg)	
D3.	Body mass index (in kg/m^2^)	
D4.	Blood pressure (in mm of Hg)	Reading 1; Reading 2; Reading 3
D5.	Capillary blood sugar (mg/dL)	
D6.	Blood cholesterol (if reports available) (in mmol/L)	
E.	WHO ISH CVD risk score	Score ____ Color zone ___ Risk zone ___
	Signature Date	

**Table 12:** Questionnaire Part II (diet history) ^*^1 kachori = 150 g ^**^1 cup = 100 mL ^***^Mention how many times daily, weekly, or monthly The dietary component of the questionnaire was adapted from the semiquantitative Food Frequency Questionnaire developed and validated for urban and rural Indian populations by Bowen et al. [14], complemented by nutrient values derived from the Indian Food Composition Tables 2017, given in the Dietary Guidelines for Indians 2024 published by the ICMR-National Institute of Nutrition [1]. The questionnaire was pilot tested to ensure clarity and feasibility ICMR: Indian Council of Medical Research

Part II					
F. Diet and food habits					
Food item	Amount	How often do you take (in the last 12 months)?^***^			
	(in grams/mL per intake)^*^	Daily	Weekly	Monthly	Less than monthly/never
Salad (e.g., cucumber/tomato/carrot/onion, etc.: uncooked preparation)					
Green leafy vegetables (e.g., spinach, fenugreek, etc.)					
Other vegetables (e.g., bhidi (okra)/lauki/parwal/gourds, etc.: usually cooked)					
Fruits (e.g., apple/guava/pomegranate/orange, etc.)					
Nuts and raisins (e.g. cashews, almonds, etc.)					
Milk^**^					
Milk products (e.g., curd and paneer)					
Beverages^**^ (e.g., tea/coffee)					
Cold drinks/sugar-sweetened drinks (e.g., Coca-Cola, juices)					
Packaged eatables (e.g., biscuits)					
Oily/fried snacks (e.g., samosa, kachori)					
Meat/fish/eggs					
Cereals (e.g., rice/wheat/millets)					

Appendix 2

**Table 13:** Average daily macronutrient values obtained from the amount of food consumed in some food groups among the study participants (n = 400) The average daily macronutrient intake from various food groups for each individual were further compounded using nutrient charts of Indian Food Composition Tables 2017, given in the Dietary Guidelines for Indians 2024 published by the ICMR-National Institute of Nutrition [1] SD: standard deviation; ICMR: Indian Council of Medical Research

Food group	n	Average daily intake (g/d)	Average daily nutrient values (cardioprotective foods), mean (SD)							
			Carbohydrate (g/d)	Energy (Kcal/d)	Protein (g/d)	Fat (g/d)	Calcium (mg/d)	Magnesium (mg/d)	Iron (mg/d)	Zinc (mg/d)
Salad	199	35.0 (37.9)	1.9 (2.0)	10.6 (11.4)	0.4 (0.4)	0.1 (0.1)	7.2 (7.9)	6.0 (6.5)	0.1 (0.1)	0.0 (0.1)
Green leafy vegetables	334	32.5 (22.8)	1.6 (1.1)	14.6 (10.3)	1.2 (0.9)	0.2 (0.2)	90.7 (63.8)	11.6 (8.2)	2.6 (1.8)	0.1 (0.1)
Other Vegetables	390	105.4 (66.6)	5.3 (3.3)	36.9 (23.3)	1.9 (1.2)	0.4 (0.3)	40.2 (25.4)	22.5 (14.2)	1.0 (0.6)	0.2 (0.1)
Fruits	251	59.4 (71.7)	6.5 (7.9)	35.1 (42.3)	0.6 (0.7)	0.4 (0.4)	16.8 (20.2)	6.1 (7.4)	0.4 (0.4)	0.1 (0.1)
Nuts and raisins	112	1.9 (2.7)	0.3 (0.5)	9.7 (13.9)	0.3 (0.5)	0.8 (1.1)	4.0 (5.7)	3.5 (5.0)	0.1 (0.2)	0.0 (0.1)
Milk	97	42.5 (51.0)	2.1 (2.6)	30.6 (36.8)	1.3 (1.6)	1.8 (2.1)	54.2 (65.1)	-	0.1 (0.1)	0.1 (0.1)
Milk products	217	22.8 (26.1)	3.6 (4.2)	76.8 (87.8)	4.9 (5.6)	4.2 (4.8)	172.0 (196.8)	1.7 (1.9)	0.4 (0.5)	0.1 (0.1)

**Table 14:** Average daily micronutrient values obtained from the amount of food consumed in some food groups among the study participants (n = 400) The average daily dietary fiber and micronutrient intake from various food groups for each individual were further compounded using nutrient charts of Indian Food Composition Tables 2017, given in the Dietary Guidelines for Indians 2024 published by the ICMR-National Institute of Nutrition [1] SD: standard deviation; ICMR: Indian Council of Medical Research

Food group	n	Average daily nutrient values (cardioprotective foods), mean (SD)								
		Dietary fiber (g/d)	Vitamin B1 (mg/d)	Vitamin B2 (mg/d)	Vitamin B3 (mg/d)	Vitamin B6 (mg/d)	Vitamin B9 (mcg/d)	Vitamin C (mg/d)	Vitamin A (mcg/d)	Vitamin D (mcg/d)
Salad	199	0.9 (1.0)	0.0 (0.0)	0.0 (0.0)	0.1 (0.1)	0.0 (0.0)	7.8 (8.5)	4.1 (4.4)	92.4 (100.1)	1.3 (1.4)
Green leafy vegetables	334	0.6 (0.5)	19.5 (13.7)	41.5 (29.2)	202.9 (142.6)	31.7 (22.3)	10.3 (7.2)	14.8 (10.4)	129.3 (90.9)	1.1 (0.8)
Other vegetables	390	2.1 (1.3)	43.5 (27.5)	45.9 (29.0)	385.0 (243.1)	102.8(64.9)	30.1 (19.0)	24.9 (15.7)	19.4 (12.2)	2.5 (1.6)
Fruits	251	1.2 (1.4)	20.7 (24.9)	12.9 (15.6)	219.7 (264.9)	38.7 (46.6)	6.8 (8.2)	21.8 (26.3)	21.1 (25.4)	2.2 (2.6)
Nuts and raisins	112	0.2 (0.2)	7.4 (10.5)	2.6 (3.8)	60.6 (86.6)	5.9 (8.4)	0.9 (1.3)	0.0 (0.0)	0.0 (0.0)	0.2 (0.2)
Milk	97	-	34.0 (40.8)	34.0 (40.8)	59.5 (71.5)	6.8 (8.2)	1.3 (1.6)	1.4 (1.7)	7.3 (8.8)	0.2 (0.3)
Milk products	217	-	28.5 (32.6)	110.3 (126.3)	62.6 (71.7)	1.7 (2.0)	2.7 (3.1)	0.3 (0.4)	17.4 (19.9)	0.0 (0.0)

**Table 15:** Average daily macronutrient, sugar and sodium values obtained from the amount of food consumed in rest of the food groups among the study participants (n = 400) ^*^Standard cup size taken as 100 mL. One 100 mL cup of tea = 37.5 Kcal. The average daily macronutrient and micronutrient intake from various food groups for each individual were further compounded using nutrient charts of the Indian Food Composition Tables 2017, given in the Dietary Guidelines for Indians 2024 published by the ICMR-National Institute of Nutrition [1] SD: standard deviation; ICMR: Indian Council of Medical Research

Food group	n	Average daily intake (g or mL/d)	Average daily nutrient values (cardiorisk foods), mean (SD)								
			Carbohydrate (g/d)	Energy (Kcal/d)	Protein (g/d)	Fat (g/d)	Calcium (mg/d)	Magnesium (mg/d)	Total sugar (/d)	Added sugar (/d)	Sodium (mg/d)
Meat/fish/egg	275	21.8 (16.3)	-	54.5 (40.8)	4.5 (3.4)	1.5 (1.1)	4.1 (3.0)	2.5 (1.9)	-	-	-
Oily/fried items	244	16.7 (21.2)	3.8 (4.8)	60.5 (76.6)	0.7 (0.9)	4.7 (5.9)	3.9 (4.9)	-	-	-	-
Packaged items	261	14.7 (13.5)	11.4 (10.5)	66.7 (61.5)	1.0 (0.9)	1.9 (1.8)	-	-	3.7 (3.5)	3.7 (3.4)	43.5 (40.1)
Cold/sweet drinks	126	39.6 (62.0)	4.3 (6.8))	17.4 (27.3)	-	-	-	-	4.2 (6.6)	4.2 (6.6)	3.4 (5.3)
Tea*	329	206.3 (124.6)	-	77.3 (46.7)	-	-	-	-	-	-	-

**Table 16:** Average daily micronutrient values obtained from the amount of food consumed in rest of the food groups among the study participants (n = 400) ^*^Standard cup size taken as 100 mL. One 100 mL cup of tea = 37.5 Kcal. The average daily macronutrient and macronutrient intake from various food groups for each individual were further compounded using nutrient charts of the Indian Food Composition Tables 2017, given in the Dietary Guidelines for Indians 2024 published by the ICMR-National Institute of Nutrition [1] SD: standard deviation; ICMR: Indian Council of Medical Research

Food group	n	Average daily nutrient values (cardiorisk foods), mean (SD)									
		Iron (mg/d)	Zinc (mg/d)	Vit B1 (mg/d)	Vit B2 (mg/d)	Vit B3 (mg/d)	Vit B6 (mg/d)	Vit B9 (mcg/d)	Vit C (mg/d)	Vit A (mcg/d)	Vit D (mcg/d)
Meat/fish/egg	275	0.3 (0.2)	0.4 (0.3)	17.8 (13.3)	23.8 (17.8)	604.0 (452.1)	47.9 (35.9)	1.2 (0.9)	-	0.4 (0.3)	0.2 (0.2)
Oily/fried items	244	0.2 (0.3)	0.1 (0.2)	-	-	-	-	2.0 (2.5)	2.1 (2.7)	-	-
